# Normalization of lymphocyte count after high ablative dose of I-131 in a patient with chronic lymphoid leukemia and secondary papillary carcinoma of the thyroid. Case report

**DOI:** 10.1590/S1679-45082014RC2657

**Published:** 2014

**Authors:** Anneliese Rosmarie Gertrud Fischer Thom, Nelson Hamerschlak, Verônica Goes Teles, Akemi Osawa, Fabio Pires de Souza Santos, Denise da Cunha Pasqualin, Jairo Wagner, Lilian Yuri Itaya Yamaga, Marcelo Livorsi da Cunha, Guilherme de Carvalho Campos, Marcelo Buarque de Gusmão Funari

**Affiliations:** 1 Hospital Israelita Albert Einstein, São Paulo, SP, Brazil.; 2 Sociedade Brasileira de Diabetes, São Paulo, SP, Brazil.

**Keywords:** Lymphoma, Lymphocyte count, Leukemia, Thyroid neoplasms/therapy, Iodine radioisotopes/therapeutic use, Case reports

## Abstract

The authors report the case of a 70-year-old male patient with chronic lymphoid leukemia who presented subsequently a papillary carcinoma of the thyroid with metastases to regional lymph nodes. The patient was treated with surgical thyroidectomy with regional and cervical lymph node excision and radioiodine therapy (I-131). The protocolar control scintigraphy 4 days after the radioactive dose showed I-131 uptake in both axillae and even in the inguinal regions. PET/CT showed faint FDG-F-18 uptake in one lymph node of the left axilla. An ultrasound guided fine needle biopsy of this lymph node identified by I-131 SPECT/CT and FDG-F-18 PET/CT revealed lymphoma cells and was negative for thyroid tissue and thyroglobulin content. The sequential blood counts done routinely after radiation treatment showed a marked fall until return to normal values of leucocytes and lymphocytes (absolute and relative), which were still normal in the last control 19 months after the radioiodine administration. Chest computed tomography showed a decrease in size of axillary and paraaortic lymph nodes. By immunohistochemistry, cells of the lymphoid B lineage decreased from 52% before radioiodine therapy to 5% after the procedure. The authors speculate about a possible sodium iodide symporter expression by the cells of this lymphoma, similar to some other non-thyroid tumors, such as breast cancer cells.

## RESUMO

Os autores relatam o caso de um paciente de 70 anos com leucemia linfóide crônica que apresentou subsequentemente um carcinoma papilífero da tireóide com metástases para linfonodos regionais. O paciente foi tratado com tireoidectomia total cirúrgica com exérese de linfonodos regionais e cervicais e radioiodoterapia (I-131). A pesquisa de corpo inteiro protocolar de controle 4 dias após a dose radioativa mostrou captação de I-131 em ambas as axilas e mesmo nas regiões inguinais. PET/CT mostrou discreta captação de FDG-F-18 em um linfonodo da axila esquerda. A biópsia por agulha fina guiada por ultrassom deste linfonodo identificado por SPECT/CT com I-131 e PET/CT com FDG-F-18 revelou células linfomatosas e foi negativa para tecido tireoidiano e conteúdo de tireoglobulina. Os hemogramas sequenciais feitos rotineiramente após tratamento com radiações mostraram uma acentuada queda até retorno aos valores normais de leucócitos e de linfócitos (absolutos e relativos), que continuavam normais no último controle 19 meses após a administração do radioiodo. Tomografia computadorizada de tórax mostrou uma redução em tamanho de linfonodos axilares e para-aorticos. Por imunohistoquímica, as células da linhagem linfoide B decresceram de 52% antes da radioiodoterapia para 5% depois do procedimento. Os autores conjeturam sobre uma possível expressão de *symporter* de iodeto de sódio pelas células deste linfoma, à semelhança de outros tumores não tireoidianos, tais como células de câncer da mama.

## INTRODUCTION

It is well known that chronic lymphocytic leukemia/small cell lymphocytic lymphoma (CLL/SCLL) predispose to the occurrence of subsequent neoplasms, mainly kidney and skin cancers.^(1–7)^ The association with thyroid carcinoma is extremely rare, according to the medical literature.^(8, 9)^

We present the case of a patient with chronic lymphocytic leukemia and secondary papillary thyroid carcinoma whose primary neoplasm had an unusual evolution after a single therapeutic dose of I-131 intended to ablate thyroid remnants after total thyroidectomy.

## CASE REPORT

I.F., a 70-year-old male patient, was diagnosed as having chronic lymphocytic leukemia (CLL) in July 2009. During the staging workup, a huge substernal multinodular goiter, with an estimated volume of 82cm^3^ was found by computed tomography (CT). An ultrasound (US) guided fine needle biopsy of one of the nodules was suspicious for thyroid cancer. This suspicion was reinforced by a serum thyroglobulin value of 936.9ng/mL (chemiluminescence immunoassay), measured in October of the same year.

As for the CLL, no specific treatment had been recommended. However, in view of the above mentioned findings concerning the thyroid, the patient was submitted to total thyroidectomy with regional lymph node (LN) resection in January 5, 2010. A total of 29 LN were excised (5 right paratracheal, 8 from level VI, 5 from level VII on the right side, 7 from level VI and 4 from level VII on the left side).

The pathological diagnosis of the excised material reported: “follicular variant of papillary microcarcinoma of the thyroid in the right lobe, confined to the thyroid gland, measuring 1.2mm, without involvement of surgical margins, extrathyroid tissue, or vascular invasion; multinodular goiter with foci of lymphocytic thyroiditis”.

Metastases were found in 8/29 LN: in all 5 right paratracheal LN and in 3/8 LN of level VI, on the right site. All others were free of disease.

The patient was referred for internal radiation therapy with I-131, which was planned for February 2010 and was preceded by a whole-body scintigraphy (WBS) one week before. Preparation for WBS and subsequent treatment was instituted during one month according to the international guidelines. The patient had remained without hormone replacement since the surgery.

In the meantime the following follow-up studies were done, listed with the respective results:

– Chest X-ray: normal.
– US of the cervical region: enlarged LN in levels I, II, and VI bilaterally consistent with an inflammatory reaction.
– CT of the chest and abdomen: enlarged axillary and retroperitoneal LN, not further investigated and attributed to the underlying lymphocytic leukemia.

By that time (January 27), his complete blood count showed 4.74x10^6^ erythrocytes, 19,200 leukocytes from which 13,958 (72.7%) were lymphocytes, and 265,000 platelets.

Serum values obtained in January 23 were: TSH: 63mIU/L; thyroglobulin: 93.4ng/mL (963.9ng/mL before surgery); anti-thyroglobulin antibodies: absent.

**Figure 1:**
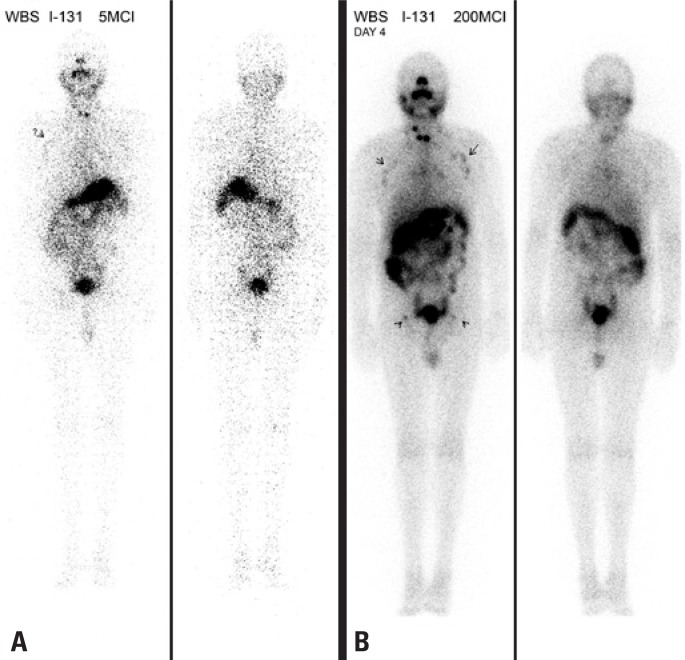
Whole-body scintigraphy before (1A) and 4 days after (1B) therapeutic dose of I-131. Uptake in axillae is suspected in the pre-dose scan but is evident in the post-dose scan. Note uptake also in the inguinal regions

I-131 WBS done on January 27, 2010 detected tiny remnants of iodine avid tissue in the anterior cervical region, with 24 hour uptake <1% of the tracer dose. No other definite uptake was seen besides a very faint dubious higher concentration in both axillary regions, which was disregarded (Figure 1A).

A therapeutic dose of 7,400MBq (200mCi) I-131 was administered on February 2, 2010.

**Figure 2:**
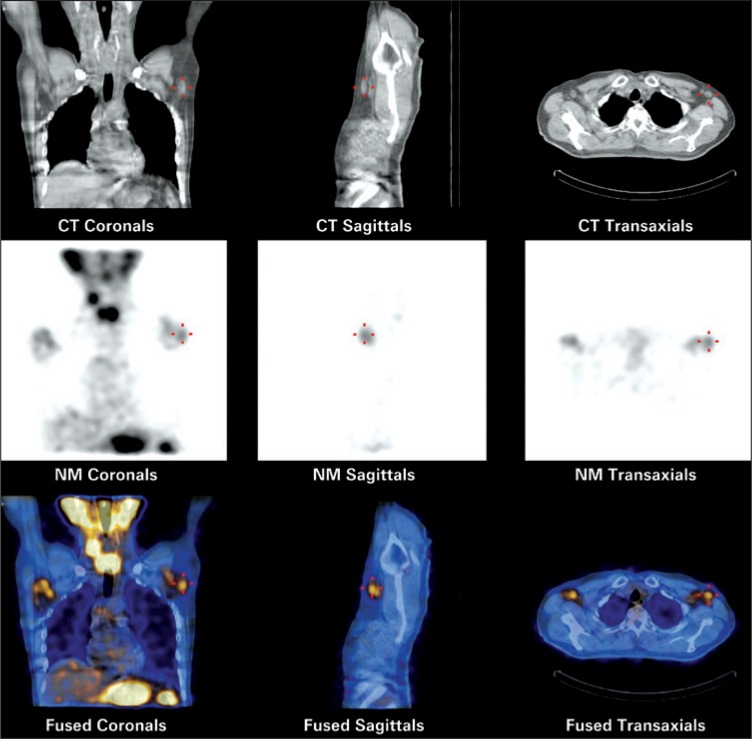
SPECT/CT 4 days after I-131 therapy. Beside radioiodine concentration in the surgical remnants of the thyroidectomy, significant uptake is detected in the axillary lymph nodes, mainly in the left axillat axilla

At the routine post-therapeutic WBS done on February 6, in addition to the expected uptake in the cervical remnants, definite uptake in both axillary regions and even in tiny structures in the inguinal regions was seen (Figure 1B). The planar scintigrams were complemented by a SPECT/CT study of the chest, which clearly showed radioiodine concentration in the axillary lymph nodes. At least one enlarged lymph node in the left axilla could be precisely identified (Figure 2).

Metastatic spread of thyroid carcinoma to the axillary lymphatic chains is a very rare condition and is generally related to advanced disease according to the literature,^(10–13)^ which was not the case of our patient. Another potential mechanism mentioned in the literature is an anomalous lymphatic drainage caused by a complex surgery,^(10)^ which could have been possible in that case.

On the other hand, the moderate diffuse and bilateral enlargement of the lymph nodes found upon clinical examination was more consistent with lymphoma. A differential diagnosis was imperative for decision-making between surgical or conservative treatment.

A FDG-F-18 PET/CT revealed only faint metabolic activity in both axillae, slightly more pronounced (SUVmax 1.3) in one nodule on the left side, assumed to be the same that showed the highest radioiodine uptake.

Because the patient refused open biopsy, a SPECT/CT, PET/CT, and ultrasound-guided fine needle aspiration biopsy of the well-defined left axillary LN was performed. The aspirated material was submitted to cytology, flow cytometry, and dosing of thyroglobulin and anti-thyroglobulin antibodies.

Cytology analysis reported the absence of epithelial cells and calcification and was consistent with CLL/SCLL.

Flow cytology/immunophenotypic profile showed CD19+ cells with coexpression of CD5, CD20, CD23 and kappa light chain, being also consistent with CLL/SCLL.

Thyroglobulin and anti-thyroglobulin antibodies measured in the biopsied tissue by chemiluminescence immunoassay were undetectable.

Based on these results, thyroid carcinoma metastases were ruled out and adenomegaly by CLL/SCLL was reported.

**Figure 3:**
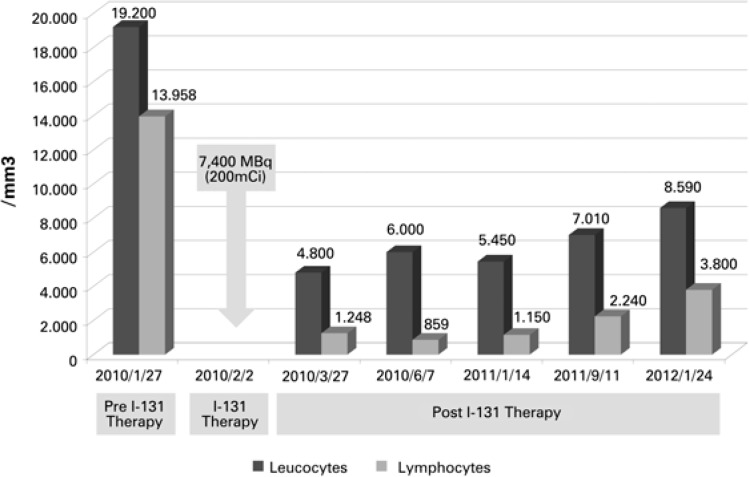
Graphic representation of the sequential leukocyte and lymphocyte counts before and along 19 months after internal radiation therapy with I-131

As part of the follow-up protocol of radioiodine treatment, sequential blood counts were done weekly between the 3rd and 6th week and eventually at later times after the radioactive dose. Although a transient drop of white blood cells, and particularly lymphocytes, always occurs after radiation therapy, an unusual reduction of the number of leucocytes mainly due to the drop of lymphocytes was seen in this patient. There was a remarkable reduction in the absolute as well as in the relative number of lymphocytes, which returned to normal values and remained normal for up to 19 months (September 2011). In January 24, 2012, leukocyte count was 8,590/mm^3^ (100%) and lymphocytes were 3,800/mm^3^ (44%), showing a small rise in the relative count beyond the normal upper limit of 40% (Table 1, Figure 3).

**Table 1:** Erythrocyte, leucocyte, lymphocyte and platelet counts in sequential hemograms from immediately before radioiodine therapy until 19 months after the treatment

Date	6 days prior I-131	I-131 therapy	Time after I-131 therapy						
	Jan 27, 2010	Feb 2, 2010	Mar 6, 2010 4 week	Mar 27, 2010 7 week	Jul 6, 2010 5 month	Oct 20, 2010	Jan 14, 2011	Sep 17, 2011	Jan 24, 2012
Erythrocytes /mm^3^	4.74x10^6^	7,400mBq	4.32x10^6^	3.87x10^6^	4.08X10^6^	4.54x10^6^	4.28x10^6^	4.58X10^6^	4.58X10^6^
Leucocytes/mm^3^	19,000	(200 mCi)	5,730	4,800	6,000	5,190	5,450	7,010	8,590
Lymphocytes/mm^3^	13,958		1,250	1,248	859	1,520	1,150	2,240	3,800
Lymphocytes relative count	72%		21%	26%	14.3%	29.3%	27.7%	32%	44%
Platelets/mm^3^	265,000		104,000	145,000	201,000	198,000	204,000	212,000	

A flow cytometry of the peripheral blood had been performed on September 8, 2009 (before surgery), and was repeated on April 15, 2010 (2 months after I-131). Cells of the lymphoid B lineage (lymphoid B cells) dropped from 52% to 5% respectively.

**Figure 4:**
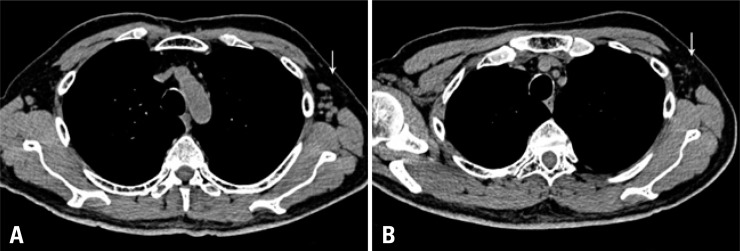
Computed tomography before (4A) and 9 weeks after (4B) I-131 therapy. Note the marked reduction of lymph nodes in the left axilla

Follow-up CT of the chest was also done on April 15, 2010, and compared to the CT from September 8, 2009, and showed a decrease in the number and the dimensions of the axillary lymph nodes (Figure 4), as well as absence of mediastinal and hilar lymphadenomegaly.

A WBS with I-131 under recombinant TSH (rhTSH) was repeated for follow-up control on February 3, 2011, and was negative for thyroid iodine-avid tissues. Serum TSH, thyroglobulin and antithyroglobulin antibodies measured before the first dose and 48 hours after the second dose of rhTSH yielded, respectively, the following results: TSH <0.05mIU/L and 306mIU/L; thyroglobulin, undetectable at both measurements; anti-thyroglobulin antibodies <5U/mL at both determinations.

## DISCUSSION

The occurrence of secondary tumors in patients with chronic lymphocytic leukemia is not infrequent and is well-documented. According to the literature, those that occur most often are kidney cancer^(3,4)^ and melanoma, among others. Secondary thyroid tumors seem to be rare, as they are found in the literature only as case reports.^(8, 9)^

Our patient presented a well-differentiated papillary microcarcinoma follicular variant secondary to (or concomitant with) chronic lymphocytic leukemia with regional LN metastases.

The finding of radioiodine uptake in the axillary lymph nodes was perplexing, since this region normally has no anatomical connection with the vessels departing from the thyroid region. References concerning axillary LN metastases of thyroid tumors are equally limited to case reports, in general of patients with advanced disease.^(10–13)^ One possible manner for this unusual spread could have been the large surgery to remove the intrathoracic goiter, as already mentioned.

As a matter of fact, FDG-F-18 PET/CT was not properly indicated for differential diagnosis because both types of tumors (CLL/SCLL and papillary thyroid carcinoma) have a rather low metabolic activity. It only ruled out a more aggressive process.

Ideally, an open biopsy with specimen histology should have been done. Bocian et al.^(8)^ published a case of coinciding metastases of papillary carcinoma and of SCLL in cervical lymph nodes. Reid-Nicholson et al.^(9)^ reported the finding of concurrent papillary thyroid carcinoma and CLL/SCLL in a thyroid nodule. A similar concurrence could explain the radioiodine uptake in the axillary LN of our patient. But one may accept that the fine-needle aspiration biopsy was informative in that it confirmed the presence of lymphoma cells by histology and immunohistochemistry, and was entirely negative for cells of the thyroid lineage as well as for thyroid cell-related substances.

Beside the contents of the material of the aspiration biopsy taken from a LN that had stored the radioactive isotope, one may hypothesize that there was, indeed, a radioiodine uptake by the lymphoma cells and a consequent radiotherapeutical action because of three additional observations: the drastic drop in white blood cell count, in particular of the lymphocytes which returned to normal values in the sixth-week follow-up after the therapeutic radioiodine dose and remained normal for up to 19 months, becoming only minimally elevated after 24 months; the reduction in size of the axillary and mediastinal LN documented by CT prior to and after radioiodine administration, and the compared blood immunohistochemistry.

Lymphoma cells are sensitive to the energy of the beta emission of I-131.^(14)^ This is the basis of radioimmunotherapy, where the antibody is the carrier of the radioisotope. But no reference to free radioiodine uptake by lymphoma cells was found in the literature.

Iodide uptake by thyroid follicular cells and cells of differentiated thyroid carcinoma is mediated by the sodium iodide symporter (NIS), which is responsible for an active co-transport mechanism of the ion across the basolateral membrane.^(15)^

NIS is also known to be expressed by breast cancer^(16)^ and by glioma tumor cells.^(17)^ One may speculate that lymphoma cells eventually might also be able to express NIS and that this might have been a possibility in our patient.

The documentation of this case is limited by the absence of a histological evaluation of the axillary lymphnode which had concentrated the radioactive agent. Nevertheless the normalization of the white cell count and the reduction in size of the axillary and paraaortic lymph nodes following the therapeutic dose of I-131 suggest that there was indeed a radiotherapeutic effect of the radioiodine on the lymphoma cells. This might be a subject for further research.
